# Testing Species Assignments in Extant Terebratulide Brachiopods: A Three-dimensional Geometric Morphometric Analysis of Long-Looped Brachidia

**DOI:** 10.1371/journal.pone.0225528

**Published:** 2019-11-27

**Authors:** Natalia López Carranza, Sandra J. Carlson

**Affiliations:** Department of Earth and Planetary Sciences, University of California, Davis, CA, United States of America; Laboratoire de Biologie du Développement de Villefranche-sur-Mer, FRANCE

## Abstract

Species of terebratulide brachiopods have been largely characterized qualitatively on the basis of morphology. Furthermore, species-level morphological variability has rarely been analyzed within a quantitative framework. The objective of our research is to quantify morphological variation to test the validity of extant named species of terebratulide brachiopods, focusing on the lophophore-supporting structures—the “long loops.” Long loops are the most distinctive and complex morphological feature in terebratellidine brachiopods and are considered to be phylogenetically and taxonomically informative. We studied eight species with problematic species identities in three genera distributed in the North Pacific: *Laqueus*, *Terebratalia*, and *Dallinella*. Given how geometrically complex long loops are, we generated 3D models from computed tomography (CT) scans of specimens of these eight species and analyzed them using 3D geometric morphometrics. Our goal was to determine ranges of variation and to test whether species are clearly distinguishable from one another in morphospace and statistically. Previous studies have suggested that some species might be overly split and are indistinguishable. Our results show that these extant species of terebratellidines can be reliably distinguished on the basis of quantitative loop morphometrics. Using 3D geometric morphometric methods, we demonstrate the utility of CT beyond purely descriptive imaging purposes in testing the morphometric validity of named species. It is crucial to treat species described and named from qualitative morphology as working hypotheses to be tested; many macroevolutionary studies depend upon the accurate assessment of species in order to identify and seek to explain macroevolutionary patterns. Our results provide quantitative documentation of the distinction of these species and thus engender greater confidence in their use to characterize macroevolutionary patterns among extant terebratellidine brachiopods. These methods, however, require further testing in extinct terebratellidines, which only rarely preserve the delicate long loop in three dimensions. In addition, molecular analyses of extant terebratellidines will test the species delimitations supported by the morphometric analyses presented in this study. [Species determination; morphological variability; 3D geometric morphometrics; terebratulide brachiopods; long loops.]

## Introduction

Despite being one of the most diverse and abundant marine invertebrates in the fossil record, particularly during the Paleozoic Era, brachiopods are often neglected in neontological studies—partly due to their low diversity, lack of economic value, and the challenges associated with collecting live specimens. Nonetheless, we can take advantage of the study of extant brachiopods to answer questions that impact both neontology and paleontology, such as species determination. The aim of our study is to quantify the morphological variation of one of the most conspicuous and geometrically complex features in terebratulide brachiopods—the mineralized loop that supports the lophophore—and test the morphological validity of extant species in both the Western and Eastern North Pacific. The main questions driving our study are: Are species distinct from one another in terms of their loop morphology? Given that species of terebratulide brachiopods have been formally described based on qualitative features, how accurate are these designations when tested within a quantitative framework? How does variability compare among extant Western Pacific and Eastern Pacific species? Since long loops are highly geometrically complex, we created 3D surface models from computed tomography (CT) scans of eight species in three genera of terebratulide brachiopods (*Dallinella*, *Laqueus*, and *Terebratalia*) thought to be closely related to each other [1], and analyzed them within a 3D geometric morphometric framework.

### Brachiopods and their mineralized lophophore supports

Brachiopods, a clade of bivalved lophotrochozoans [2], are highly suitable for studying and comparing ranges of morphologic variability in both living and fossil species. Over 5,000 genera have been recognized in the fossil record based on morphology, representing approximately 15,000 species [3]. Of all species, only fewer than 3% (approximately 400 species) are still alive today, with approximately 75% classified in the order Terebratulida [4, 5].

The order Terebratulida is a clade [5–8] comprised of articulated brachiopods with endopunctate and commonly biconvex shells, which possess a typically astrophic hinge line (i.e. curved, not parallel to the hinge axis), cyrtomatodont (interlocking hook-shaped) teeth, a functional pedicle for hard-substrate attachment, and a calcareous loop—also referred to as a *brachidium*—that resorbs and remineralizes over ontogeny while it provides internal support to and positions the plectolophe lophophore in the mantle cavity [9]. The lophophore, the feeding and respiratory organ of brachiopods, is formed by a pair of symmetrical brachia (arms) bearing ciliated tentacles that surround the mouth and create inhalant and exhalant currents in the mantle cavity. Although its main functions are feeding and respiration, the lophophore has sensory functions and, in some cases, broods larvae [5, 10–15]. Lower-level classification within the order is based on both internal and external morphology [16]; however, internal morphology is considered to play a fundamental role because it is commonly thought that external shell morphology offers little resolution for classification, given the smaller number of characters available and their potentially homeomorphic nature [1, 17–21].

**Fig 1:**
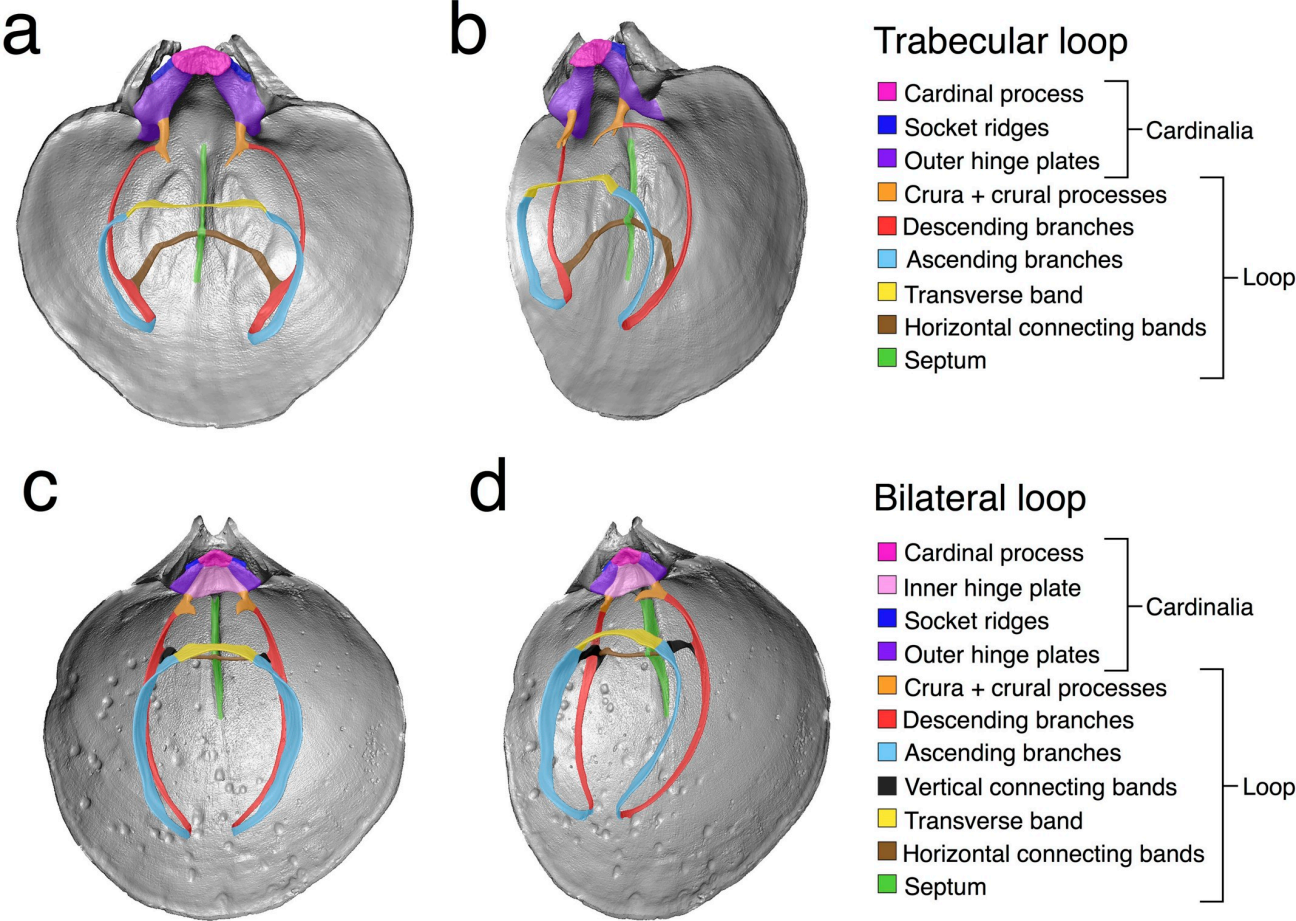
Trabecular and bilateral loops. a) Front and b) oblique view of the dorsal valve of *Terebratalia transversa* (SBMNH 616990). c) Front and d) oblique view of dorsal valve of *Laqueus vancouveriensis* (USNM PAL 716055).

Loop morphology is crucial in the taxonomy and systematics of the two suborders within Terebratulida—Terebratulidina and Terebratellidina—since they were established mainly based on variations in this character [16, 22–24]. Individuals of the suborder Terebratulidina are identified by their “short loops,” which develop exclusively from the crura (calcareous processes that extend from the posterior portion of the dorsal valve) [9, 22] and do not extend beyond half of the length of the ventral valve, while brachiopods of the suborder Terebratellidina are characterized by having “long loops” [23, 25], which extend beyond half the length of the ventral valve (Fig 1). In the remainder of this study, we will refer to long-looped brachidia as “long loops.” In general terms, long loops are composed of a pair of descending branches that extend anteriorly from the crura; these curve ventrally—forming the ascending branches—and join medially. Depending on the number and location of attachments among the various structures (i.e. descending and ascending branches, and medial septum), six terminal adult long loop types have been recognized [1].

Given our focus on North Pacific terebratellidine species, we studied two loop types—trabecular (represented in the genera *Terebratalia* and *Dallinella*, Fig 1A and 1B) and bilateral (represented in the genus *Laqueus*, Fig 1C and 1D). In both bilateral and trabecular loops the descending branches are attached to the median septum [1]; bilateral loops, additionally, possess a set of vertical connecting bands attaching the descending and ascending branches [1], which stabilize the ascending and descending branches of the lophophore, ensuring consistent separation of the inhalant and exhalant currents through the mantle cavity while feeding and respiring [14, 26].

### Species descriptions and issues surrounding species designations

*Laqueus* Dall, 1870 [27] is characterized by medium to large size, ovate and smooth shells with a rectimarginate (i.e. planar) commissure. *Laqueus erythraeus* Dall, 1920 [28] (previously referred to as *Laqueus californianus* or misspelled as *Laqueus californicus* [29]) and *L*. *vancouveriensis* Davidson, 1887 [30] potentially overlap in geographic distribution, particularly in Monterey Submarine Canyon [31]. *Laqueus vancouveriensis* had been considered as a northern subspecies of *L*. *erythraeus* (e.g. [32, 33]); however, based on its smaller valve size and larger pedicle foramen than *L*. *erythraeus*, it has been elevated to species status [29, 34, 35]. *Laqueus rubellus* (Sowerby, 1846) [36], commonly found off the coast of Japan [37, 38], is very similar in external valve morphology to *L*. *erythraeus*; however, it is considered to be a different species mostly based on their different geographic distribution and shell coloration. *Laqueus blanfordi* (Dunker, 1882) [39], also with a Japanese distribution, has oval to subpentagonal shells, with lateral sides prominently curved outwards and a truncated anterior margin. Described as similar to *L*. *blanfordi*, Yabe and Hatai [40] state that *Laqueus quadratus* differs from the latter in having a quadrate valve outline.

Although the taxonomy of *Laqueus* species has been the subject of discussion (e.g. [29, 41, 42]), names have been assigned based on qualitative assessments of valve shape and species have never been subject to further analysis, either morphological or genetic, to test their validity. It is important to note that we focused mainly on those species most commonly found in the wild and therefore more likely to be found in museum collections—*L*. *erythraeus*, *L*. *vancouveriensis*, and *L*. *rubellus*. However, twelve more extant *Laqueus* species are currently regarded as valid [43] (S1 Appendix), but with little to no information about their variability and comparability in morphology, ecology, or distribution.

The genus *Terebratalia* Beecher, 1893 [44] is characterized by medium to large shells that display a high variability in outline shape and ornamentation. *Terebratalia transversa* (Sowerby, 1846) [36], particularly known for exhibiting high ecophenotypic variability [45–48], is distributed from Alaska to Baja California [49, 50], being the only living species of the genus with a North American distribution [38, 51]. The name *Terebratalia occidentalis* (Dall, 1871) [52] has been frequently assigned to Recent specimens distributed from Monterey Bay to Baja California [49]; however, it is now distinguished as a separate genus from *Terebratalia* as *Dallinella occidentalis* (Dall, 1871) [52]. The genus *Dallinella* Thomson, 1915 [53] was established based on folding at the valve anterior—possessing rectimarginate to uniplicate shells—and the presence of trabecular loops. Therefore, *D*. *occidentalis* differs from *T*. *transversa* by having a sulcus (not a fold) on the ventral valve and a fold (not a sulcus) on the dorsal valve [35, 49]; however, the loops of *Dallinella* and *Terebratalia* are considered to be extremely similar in shape [53]. In the Western North Pacific Ocean, *Terebratalia coreanica* (Adams and Reeve, 1850) [54] shares the same general morphological characteristics with its North American congener.

When investigating fossil descriptions of *Terebratalia* species, approximately a dozen species from *Terebratalia/Dallinella* have been described for the Cenozoic of western North America and Japan [35, 51] based on slight variations in morphology (e.g. overall size and shell outline, ornamentation and ribbing, convexity). Therefore, quantifying morphological variability and recognizing what constitutes species-level variation in extant specimens is essential for determining fossil species in a more consistent and reproducible manner.

### Geometric morphometrics and 3D imaging of brachiopods

Morphological variability can be quantified using morphometric-based measurements or through geometric morphometrics. Geometric morphometrics, unlike traditional morphometrics, uses landmark coordinates to analyze changes in the geometry of morphologic structures using statistical analyses [55, 56]. Landmarks are described as anatomical loci that can be placed on a biologically or geometrically homologous point on a structure [56, 57]. Geometric morphometrics has been a useful methodology for studying brachiopod variability, particularly in the fossil record, but is not often used to test species validity (see [48, 58–64]).

With its increasing accessibility, high-resolution 3D imaging—such as CT scanning—has become a useful, non-destructive tool for the study of small, complex, and delicate internal structures like the loop and cardinalia (e.g. [65–72]). CT, in particular, ensures that shell characters crucial for identification of genera and species of articulate brachiopods (e.g. hinge teeth and plates, sockets) are preserved. Applying these imaging techniques broadens our ability to quantitatively study structures that are thought to represent a rich source of taxonomic and phylogenetic information, such as the mineralized loop.

Analyzing morphology in a quantitative manner and examining phenotypic variability in living close relatives is an essential first step to be able to test the assumption that extant and fossil species are comparable evolutionary entities. This study represents an effort to work at the species level in neontology with clear implications for paleontology, where genera are commonly treated as proxies for species [73], by approaching species as hypotheses to be tested.

## Materials & methods

**Fig 2:**
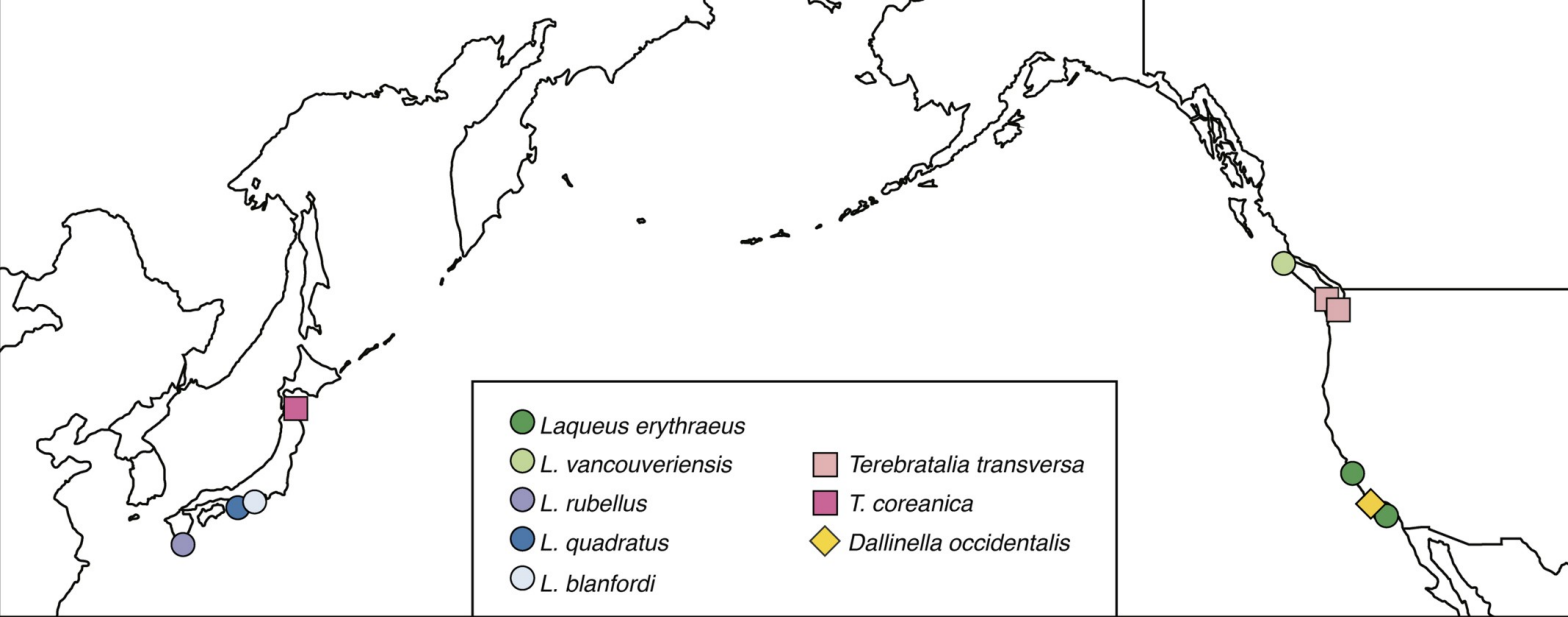
Map of specimen localities. For more detailed information see S1 Table.

To analyze long loop variability in terebratulide brachiopods, we chose *Laqueus*, *Terebratalia*, and *Dallinella* as exemplar genera. Of these three genera, we focused on a total of 58 adult individuals of the following species: *Laqueus erythraeus*, *L*. *vancouveriensis*, *L*. *rubellus*, *L*. *quadratus*, *L*. *blanfordi*, *Terebratalia transversa*, *T*. *coreanica*, and *Dallinella occidentalis* (Table 1). The localities where the specimens were originally collected are shown in Fig 2. For detailed information on repositories, specimen numbers, and localities see S1 Table. Since the samples for this study consisted of non-living specimens housed in collections, no permits were required to obtain specimens. The number of individuals analyzed is small, but was dependent on abundance in the wild, and available access to specimens with internal structures preserved in museum collections. Ideally, type specimens could be analyzed for their long loop morphology; locating and imaging these delicate specimens in museum repositories can be challenging.

**Table 1:** List of species analyzed with information on loop type, geographic distribution, and number is individuals analyzed.

Species	Loop type	Geographic distribution	Number of individuals analyzed
*Laqueus erythraeus* Dall, 1920	Bilateral	Northeastern Pacific, from Alaska to Southern California, USA	16
*Laqueus vancouveriensis* Davidson, 1887	Bilateral	Northeastern Pacific, from Alaska to Washington, USA	12
*Laqueus rubellus* (Sowerby, 1846)	Bilateral	Japan	9
*Laqueus quadratus* Yabe & Hatai, 1934	Bilateral	Japan and Taiwan	2
*Laqueus blanfordi* (Dunker, 1882)	Bilateral	Northwestern Pacific, from Kyushu Island, Japan to Kamchatka, Russia	1
*Terebratalia transversa* (Sowerby, 1846)	Trabecular	Northeastern Pacific, from Alaska Peninsula to Baja California Sur, Mexico	8
*Terebratalia coreanica* (Adams & Reeve, 1850)	Trabecular	Northwestern Pacific, along the coasts of the Yellow Sea and the Sea of Japan	5
*Dallinella occidentalis* (Dall, 1871)	Trabecular	Northeastern Pacific, from Monterey Bay, CA to Baja California Sur, Mexico	5

### CT scanning

Specimens were imaged using a MicroXCT-200 scanner from Carl Zeiss X-Ray Microscopy in the Center for Molecular and Genomic Imaging (CMGI) at the University of California, Davis. Once the specimens were scanned, 3D surface models were created using the software Amira v. 6.3.0 (Thermo Scientific).

### Landmark schemes and registration

**Fig 3:**
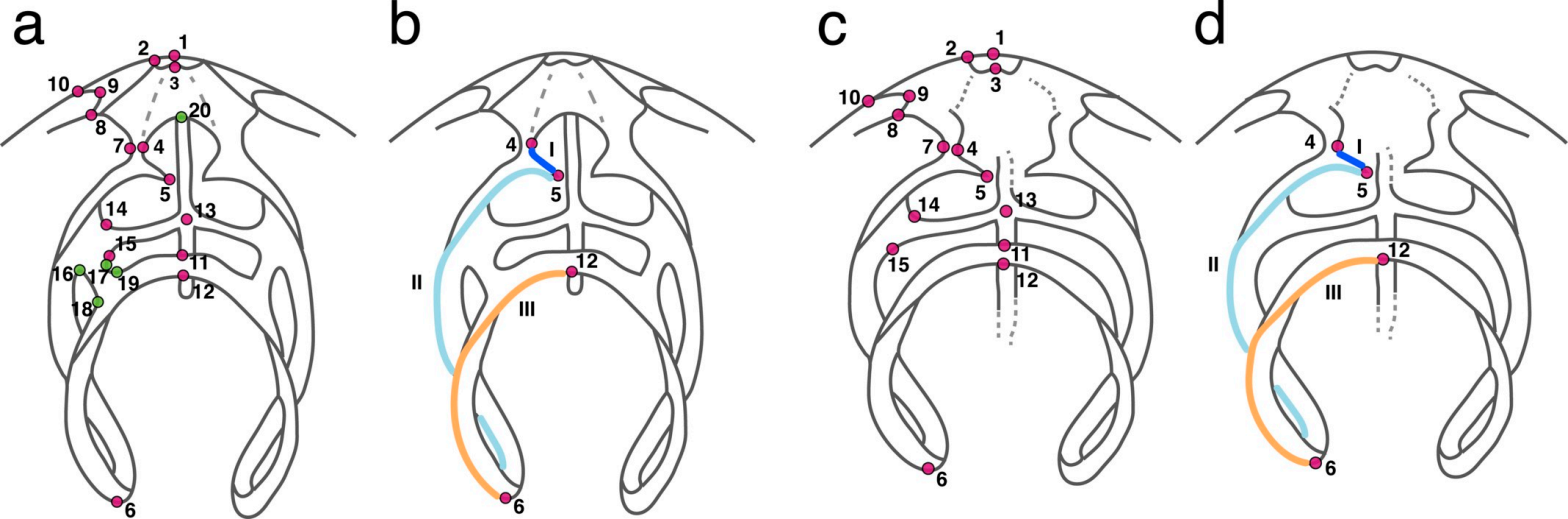
Landmark scheme based on different loop types. a) Bilateral loop with 20 landmarks and b) three curves based on semilandmarks (I-III). c) Trabecular loop with 15 landmarks and d) three curves based on semilandmarks (I-III). Landmarks in pink (1–15) represent shared landmarks between bilateral and trabecular loops; landmarks in green (16–20) represent unique landmarks found in the bilateral loop.

Depending on the loop type (bilateral and trabecular), two landmark schemes were determined (Fig 3 and S2 Table). Landmarks were selected at the junction of different structures (Type I) and at points where homology is defined by geometry (e.g. the maximal curvature or the edge of a structure, Type II) on the loop, cardinal area, and septum. Additionally, three curves were included in the analyses to capture the 3D curvature of the crus, descending and ascending branches, and transverse band. Using Stratovan Checkpoint (Stratovan Corporation), landmarks and curves were placed on the 3D surface models of the specimens. In order to determine the number of semilandmarks needed to accurately describe curve shape, we tested a dataset comprised of all 58 specimens, 15 landmarks (shared between bilateral and trabecular loops), and a total of 69 semilandmarks (a highly dense curve sampling), using the R function landmark sampling evaluation curve (LaSEC, from the package LaMBDA [74]). Using the complete dataset, LaSEC performs a generalized Procrustes analysis (GPA) of landmark coordinates and subsequently a principal component analysis (PCA). Then, it randomly subsamples three landmarks from the original dataset and performs another GPA and PCA. Using the PC plots generated from the original dataset and the subsample dataset, an ordinary Procrustes alignment is implemented and the sum of squared distances (PSS) between the two is used as a measurement of fit. With every iteration, LaSEC adds one landmark to the analysis and compares the resulting PC plot against that of the original dataset. Finally, LaSEC plots a fit trajectory (1-PSS) according to the number of landmarks that were sampled on each iteration. Ideally, the fit trajectory reaches a plateau, indicating that, regardless of the number of landmarks used, the fit remains constant. Since loops are bilaterally symmetrical, we only selected landmarks and semilandmarks on one half of the loop and shell.

### Geometric morphometric analyses

To eliminate variation due to size that is uncorrelated with shape, translation, or rotation, we superimposed our landmark configurations using a generalized Procrustes analysis [56]. Since we analyzed a combination of landmarks and semilandmarks, semilandmarks were superimposed by iteratively sliding the points to minimize bending energy between the curves. Once the Procrustes-fitted coordinates were obtained, we used exploratory data analysis, focusing on ordination methods. To explore variability according to loop type, we analyzed the following datasets: *Laqueus* (bilateral loop, n = 40; S1 Data) and *Terebratalia* and *Dallinella* (trabecular loop, n = 18; S2 Data). A principal component analysis (PCA) and a canonical variate analysis (CVA) were implemented for each of the datasets. Both PCA and CVA use Procrustes-fitted coordinates to simplify patterns of variation; however, for CVA, groups are defined *a priori*. This ordination method is useful to effectively discriminate among groups, since it rescales the axes based on within-group variation. We used CVA grouping to obtain the overall classification accuracy through a leave-one-out cross-validation. Finally, to examine the general pattern of variability, we performed a principal component analysis (PCA) on all specimens (shared landmarks of *Laqueus*, *Terebratalia*, and *Dallinella*, n = 58; S3 Data).

To test if shape is dependent on species designation and size (allometry), we performed independent Procrustes ANOVA analyses testing landmark coordinates against species identification and against centroid size, respectively. Centroid size is defined as the square root of the summed squared distances of each landmark to the centroid of the landmark configuration [56]. All geometric morphometric analyses were performed in R [75] using the packages geomorph [76] and Morpho [77].

## Results

### Landmark sampling evaluation test: How many landmarks should we include in our analyses?

**Fig 4:**
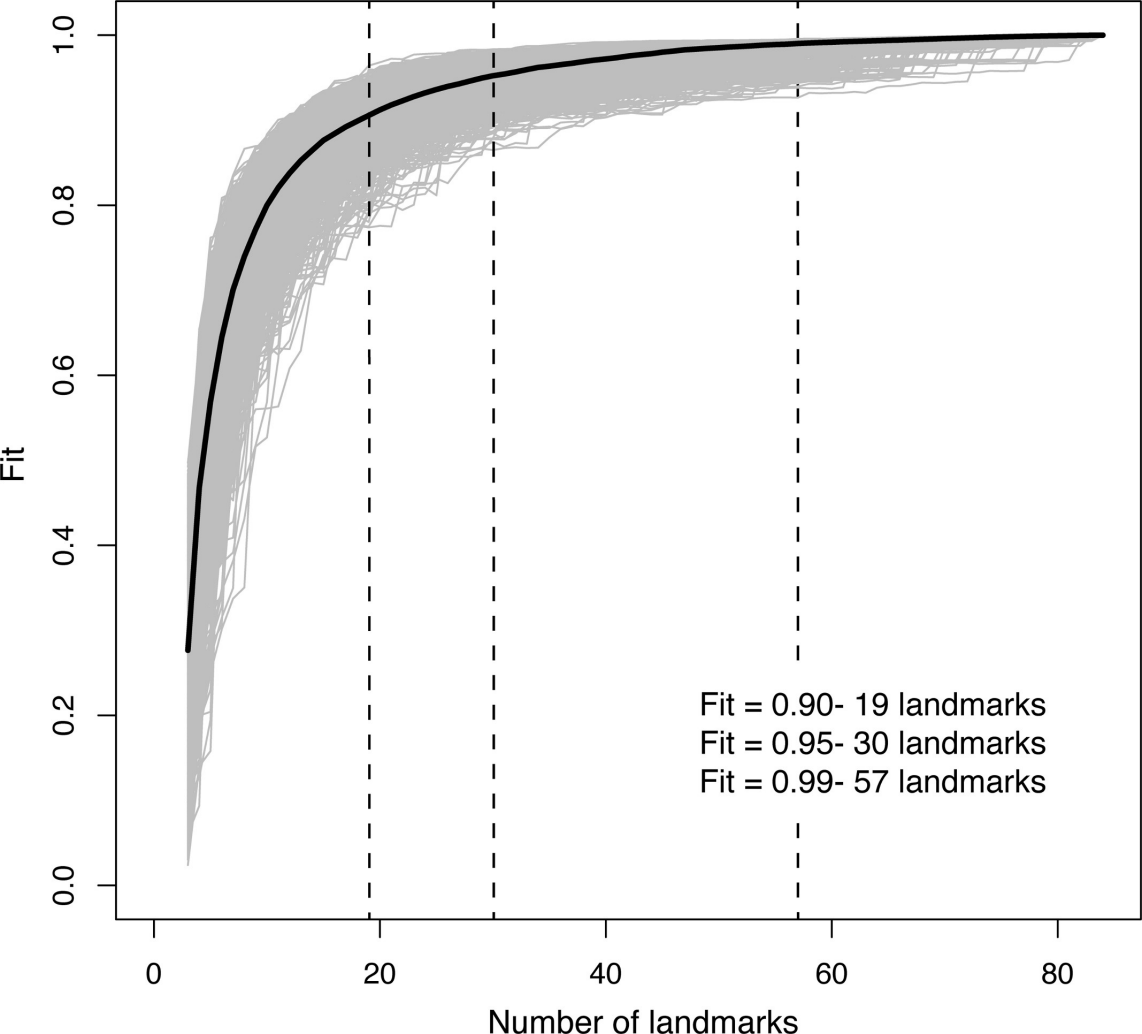
Sampling curve from landmark sampling evaluation test (LaSEC) [74] on *Laqueus*, *Terebratalia*, and *Dallinella* dataset (15 landmarks and 69 semilandmarks). A fit value of 1 indicates that no morphometric information is added when landmark number increases, providing an indication of the minimum meaningful number of landmarks. Gray lines represent iterations of subsampling; black line shows median fit corresponding to every landmark; vertical dashed black lines correspond to number of landmarks at fit values of 0.90, 0.95 and 0.99.

After performing a Landmark Sampling Evaluation test [74] on our complete dataset, we determined the appropriate number of semilandmarks to be included in our 3D geometric morphometric analyses in order to fully characterize curve shape based on the median fit. Considering our results (Fig 4) and a visual examination of our data points in 3D space, we decided to use datasets comprised of 20 and 15 landmarks for bilateral and trabecular loops respectively, and 21 semilandmarks (median fit > 0.95), therefore reducing redundancy on our landmark data.

### Variability in *Laqueus*

**Fig 5:**
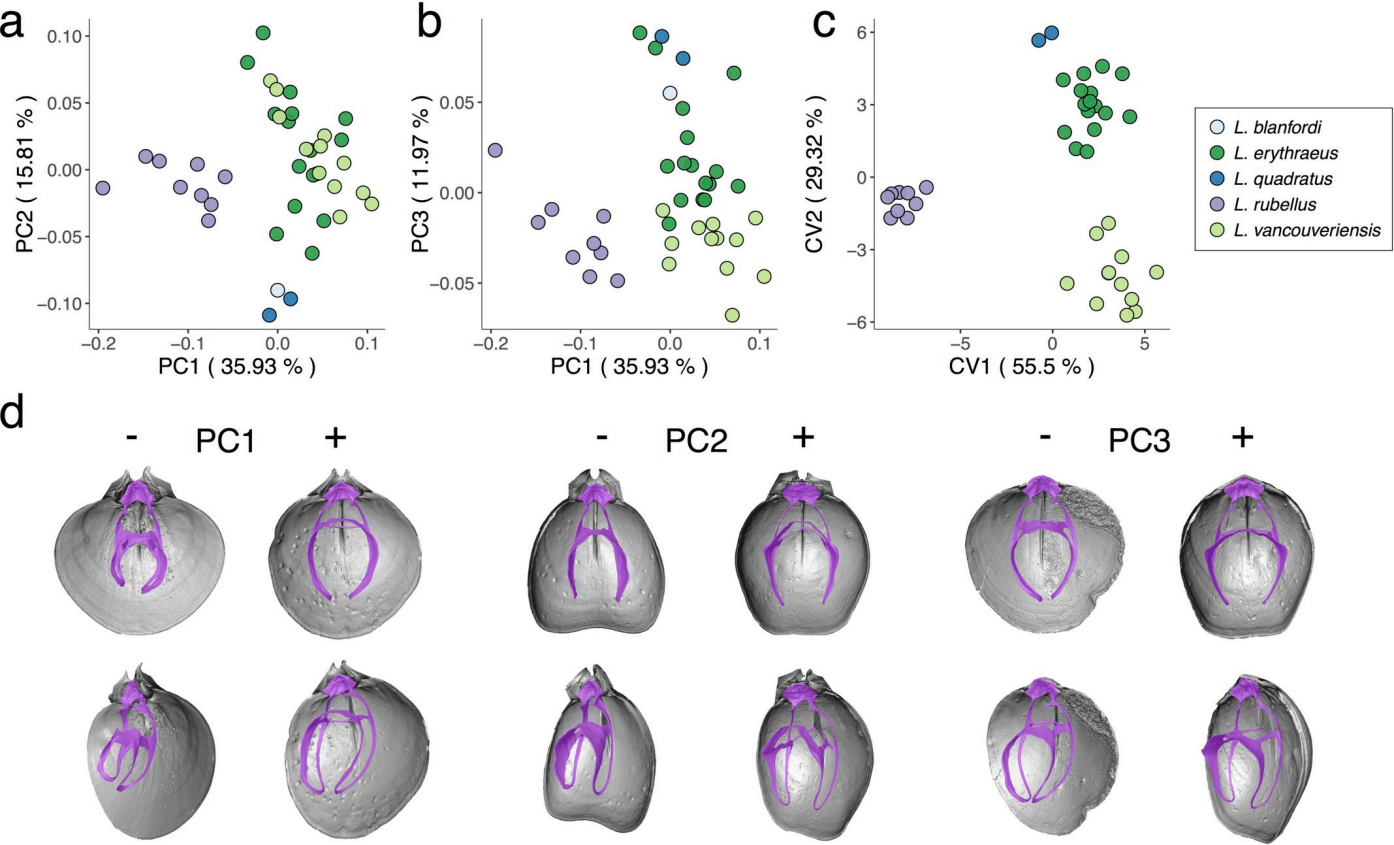
Principal component analysis (PCA) and canonical variate analysis (CVA) of *Laqueus* species with associated shape changes in loop morphology. a) PC1-PC2; b) PC1-PC3; c) CVA; d) specimens with the most negative to most positive PC score values. PC1: *L*. *rubellus* USNM PAL 7160785 (PC1-) and *L*. *vancouveriensis* USNM PAL 716055 (PC1+). PC2: *L*. *quadratus* USNM PAL 716076 (PC2-) and *L*. *erythraeus* DAV:SJCLab 0008 (PC2+). PC3: *L*. *vancouveriensis* USNM 716058 (PC3-) and *L*. *erythraeus* DAV:SJCLab 0007 (PC3+).

When analyzing individuals of the genus *Laqueus* (Fig 5), *L*. *rubellus* separates from other species along PC1 (35.93% of total variance). *L*. *erythraeus* and *L*. *vancouveriensis*, however, overlap in shape space, with *L*. *erythraeus* displaying a wider range of variability (Fig 5A). *L*. *quadratus* and *L*. *blanfordi* plot closer in morphospace to East Pacific species than to *L*. *rubellus*, separating from *L*. *erythraeus* and *L*. *vancouveriensis* along PC2. In terms of morphology (Fig 5D), from positive to negative PC1 scores, the cardinalia elongates posteriorly while the crural process elongates ventrally, and the descending and ascending branches are reduced in length. Therefore, plotted towards positive PC1 scores we observe longer loops with respect to the cardinalia; as we move towards negative values, loops and cardinalia become similar in length. In the case of PC2 (15.81% of total variance), the changes in morphology are mostly related to the length of the ascending branch, with specimens with longer ascending branches (*L*. *erythraeus* and *L*. *vancouveriensis*) plotted towards positive PC2 scores. *Laqueus erythraeus* is distinguished from *L*. *vancouveriensis* along PC3 (11.97% of total variance), only with a minimum overlap between them (Fig 5B). Specimens of *L*. *rubellus* cluster together without any overlap with other species. *L*. *quadratus* and *L*. *blanfordi* score positively along PC3. As we move towards negative PC3 scores, loops become less laterally curved and the crural process decreases in size.

Since we only had access to one scanned individual of *L*. *blanfordi*, we removed this species from the CVA, given that a leave-one-out cross-validation is also performed to test specimen classification. Our CVA plot (Fig 5C) shows that species are clearly separated in shape space, with conspecific individuals clustering together. The overall classification accuracy is 100%, with all individuals being classified correctly to their respective species (Table 2). This result is particularly relevant for *L*. *erythraeus*/*L*. *vancouveriensis*, given that there has been considerable debate as to whether they represent one or two species (see [29]). Based on loop morphology, every single individual was assigned to its named species. As part of the CVA, Mahalanobis distances were calculated (Table 2). Mahalanobis distances are an excellent way of measuring differences between groups since within-group variation is transformed isotropically, eliminating within-group variation directionality, therefore representing only the distances between group means [78]. Considering Mahalanobis distances, the two most morphologically distant species are *L*. *rubellus* and *L*. *quadratus*, while the two most similar are *L*. *erythraeus* and *L*. *vancouveriensis*. This result is consistent with our predictions; we expected the East Pacific species, *L*. *erythraeus* and *L*. *vancouveriensis*, to be the most similar in morphology, especially since they have been identified historically as a single species [29].

**Table 2:** Mahalanobis distances between species means in *Laqueus* (bold) and cross-validated classification results in percentages. Overall classification accuracy of 100%.

	*Laqueus erythraeus*	*Laqueus quadratus*	*Laqueus rubellus*	*Laqueus vancouveriensis*
*Laqueus erythraeus*	100%			
*Laqueus quadratus*	**11.482**	100%		
*Laqueus rubellus*	**11.027**	**14.141**	100%	
*Laqueus vancouveriensis*	**7.588**	**13.650**	**11.987**	[100%]

Considering the differences in overall body size between species, we tested if loop shape was dependent on size (shape ~ size) and determined that size has a statistically significant effect on loop shape in *Laqueus* (*p* = 0.001). Given this result, we tested if size and species designation (shape ~ size + species) were independent in their effects on shape using a Procrustes ANOVA. Our results indicate that size and species are dependent on each other (*p* = 0.001) and there is no common allometric component among *Laqueus* species, meaning that the pattern of shape variation with respect to size itself varies between species (i.e. different species have different patterns of shape change with regards to size). Similarly, we tested if loop shape is statistically different among species (shape ~ species) and we found that each species has a statistically significantly different loop (*p* = 0.001), indicating that, based on loop morphology alone, species of *Laqueus* are statistically distinct from one another.

### Variability in *Terebratalia* and *Dallinella*

**Fig 6:**
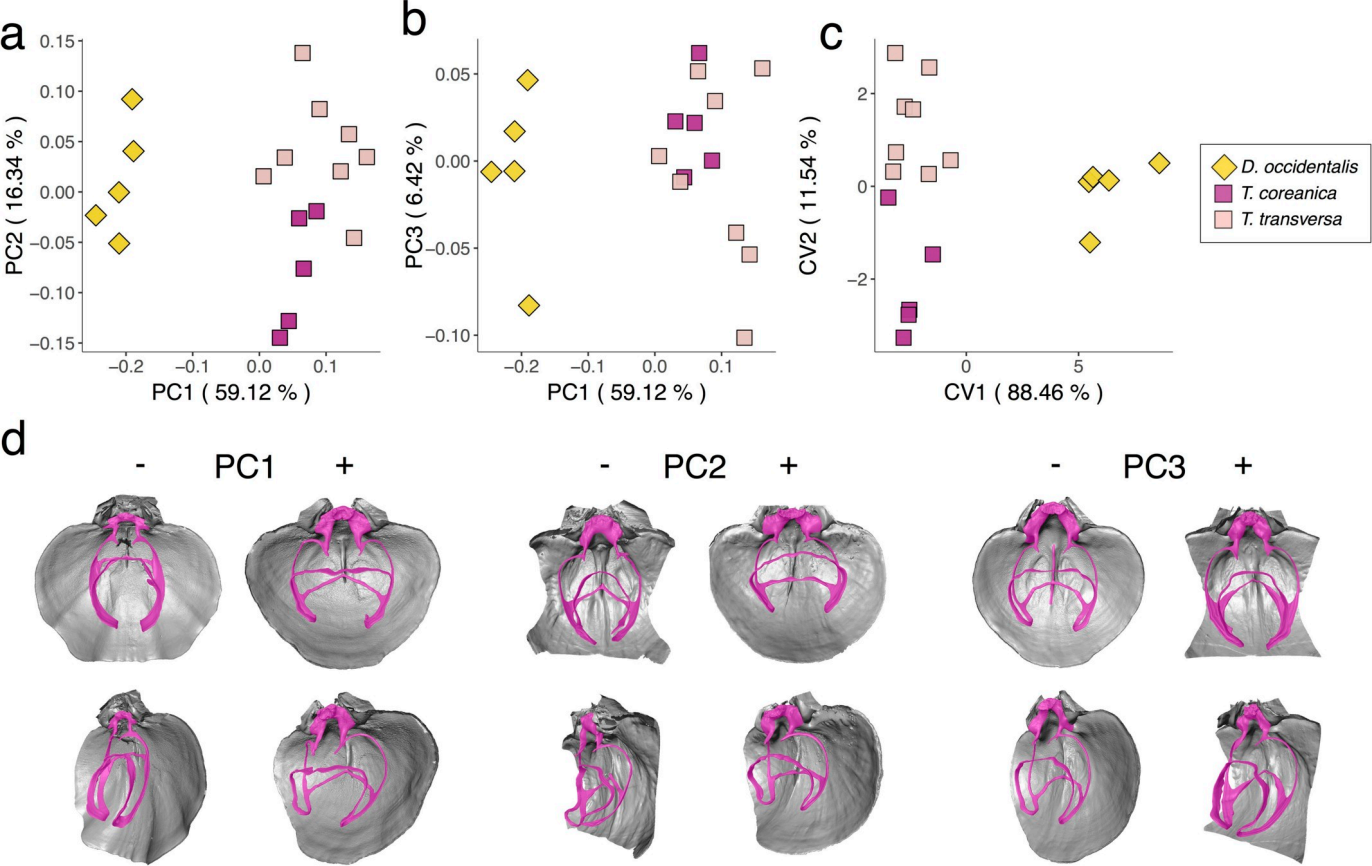
Principal component analysis (PCA) and canonical variate analysis (CVA) of *Dallinella* and *Terebratalia* species with associated shape changes in loop morphology. a) PC1-PC2; b) PC1-PC3; c) CVA; d) specimens with most negative to most positive PC score values. PC1: *D*. *occidentalis* SBMNH 467448 (PC1-) and *T*. *transversa* SBMNH 616986 (PC1+). PC2: *T*. *coreanica* USNM 716054 (PC2-) and *T*. *transversa* DAV:SJCLab 0011 (PC2+). PC3: *T*. *transversa* SBMNH 616990 (PC3-) and *T*. *coreanica* USNM 716052 (PC3+).

The PCA of *Terebratalia* and *Dallinella* distinctly separates these two genera along PC1 (59.12% of total variance), with negative PC1 scores corresponding to *Dallinella* and positive scores to *Terebratalia* (Fig 6A and 6B). Along PC2 (16.34% of total variance), individuals of *T*. *transversa* plot towards positive scores and *T*. *coreanica* towards negative. In terms of morphology (Fig 6D), from negative to positive PC1 scores, there is shortening of the descending and ascending branches and widening of the cardinalia, with *Dallinella* having more elongated loops and narrower cardinalia than *Terebratalia*. Shape change along PC2 is represented mainly by a difference in thickness—i.e. how far apart the descending and ascending branches are from each other—with *T*. *coreanica* having thinner loops than *T*. *transversa*. Individuals of *T*. *coreanica* overlap with *T*. *transversa* along PC3 (6.42% of variance; Fig 6B). Changes in morphology along PC3 correspond to a lateral widening of the loop; wider loops plot towards positive scores. In comparison to the other species analyzed, *T*. *transversa* shows a broader range of variability in terms of loop width.

After performing a CVA, which maximizes between-species differences, our plot (Fig 6C) shows clear separation between *Dallinella* and *Terebratalia* along CV1. *Terebratalia* species have similar CV1 scores but differ in CV2, without showing any overlap. However, overall classification accuracy was 72.22%. *Dallinella* specimens were classified correctly 100% of the time, while individuals of *T*. *coreanica* and *T*. *transversa* were assigned to their correct species 60% and 62.5% of the time, respectively (Table 3). Mahalanobis distances between *Dallinella* and *Terebratalia* are at least 2.5 times the interspecific distance between the two species of *Terebratalia* (Table 3).

**Table 3:** Mahalanobis distances between species means in *Dallinella* and *Terebratalia* (bold) and cross-validated classification results in percentages. Overall classification accuracy of 72.22%.

	*Dallinella occidentalis*	*Terebratalia coreanica*	*Terebratalia transversa*
*Dallinella occidentalis*	100%		
*Terebratalia coreanica*	**9.110**	60%	40%
*Terebratalia transversa*	**8.757**	**3.426** 37.5%	62.5%

When testing if shape variation is attributable to differences in size, we determined that size does not have a statistically significant impact on shape in *Terebratalia* and *Dallinella* (*p* = 0.122). Moreover, we found that species’ loops do differ in shape in a statistically significant manner (*p* = 0.001). Even though exploratory methods such as CVA showed overlap and species assignment errors between *T*. *transversa* and *T*. *coreanica*, statistical analysis using Procrustes ANOVA show that they are significantly different from one another. Considering how highly variable individuals from the genus *Terebratalia* are, it is not surprising that individuals of *T*. *transversa* and *T*. *coreanica* were occasionally assigned to the incorrect species even if statistical methods analyzing loop geometric morphometrics reliably differentiate them.

### Combined dataset: General pattern of variability and among genera and species

**Fig 7:**
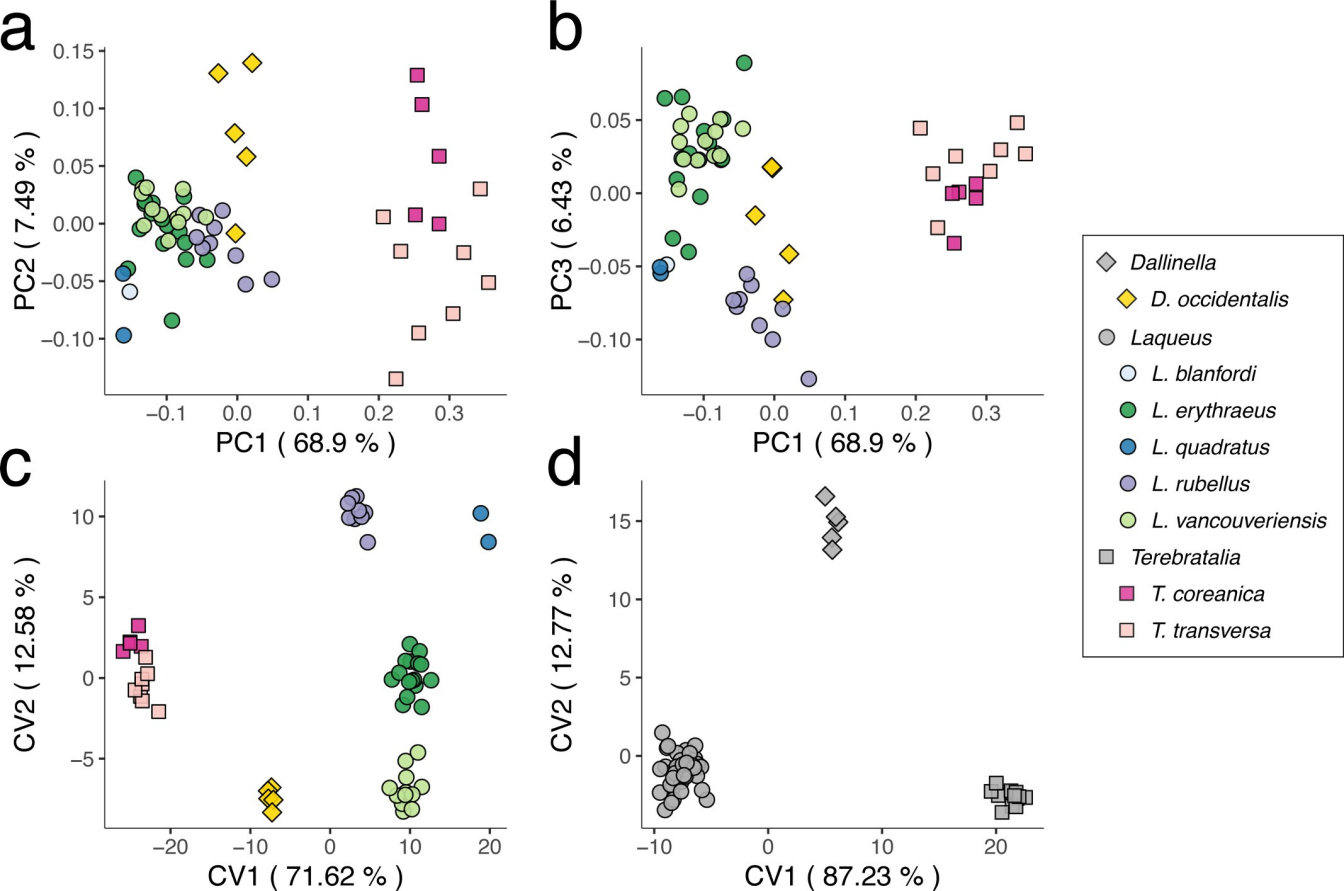
Principal component analysis (PCA) and canonical variate analyses (CVA) of a combined dataset (*Laqueus*, *Terebratalia*, and *Dallinella*). a) PC1-PC2; b) PC1-PC3; c) CVA of species; d) CVA of genera.

To explore the overall pattern of variability among specimens of *Laqueus*, *Terebratalia*, and *Dallinella* (n = 58), we analyzed a combined dataset with a total of 15 landmarks and three curves (21 semilandmarks). This dataset included shared landmarks between bilateral (*Laqueus*) and trabecular (*Terebratalia* and *Dallinella*) loops. The principal component analysis (Fig 7A and 7B) reveals that individuals from each genus tend to cluster together in shape space, although with some overlap between *Laqueus* and *Dallinella*. The first principal component (68.9% of total variance) separates *Terebratalia* from *Dallinella* and *Laqueus*. The second principal component (7.49% of total variance) separates *Laqueus* from *Dallinella*, and separates the two species of *Terebratalia* (*T*. *transversa* and *T*. *coreanica*). With respect to *Laqueus*, the third principal component (6.43% of total variance) separates the West Pacific species (*L*. *blanfordi*, *L*. *quadratus*, and *L*. *rubellus*) from the East Pacific (*L*. *erythraeus* and *L*. *vancouveriensis*).

In order to compare morphological distances among all genera and species, we performed a CVA of all individuals of *Laqueus*, *Terebratalia*, and *Dallinella*, grouping by species (Fig 7C) and genera assignments (Fig 7D). When analyzing species groupings (Fig 7C), specimens of each species form distinct clusters in shape space, with genera separating along CV1. Even though we decided to analyze the whole dataset, it is important to keep in mind that *Laqueus* has a different loop type than *Terebratalia* and *Dallinella*; therefore, morphological distances between *Laqueus* and *Terebratalia*/*Dallinella* do not fully represent the extent of morphological difference between loop types. However, these results are informative when comparing distances among congeners (Table 4). For example, Mahalanobis distance between *T*. *coreanica* (NW Pacific) and *T*. *transversa* (NE Pacific) is almost equal to that between *L*. *rubellus* (NW Pacific) and *L*. *erythraeus* (NE Pacific), 15.2 and 15.32 respectively. *Laqueus erythraeus* and *L*. *vancouveriensis* had the smallest distance among every pair of species, which is unsurprising given their geographic distribution and taxonomic history. Furthermore, biogeographically, West Pacific species (*T*. *coreanica*, *L*. *rubellus*, and *L*. *quadratus*) have positive CV2 values and separate from the more negative values of the East Pacific species.

**Table 4:** Mahalanobis distances among species means for *Dallinella*, *Laqueus*, and *Terebratalia* (bold) and cross-validated classification results in percentages. Overall classification accuracy of 100%.

	*Do*	*Le*	*Lq*	*Lr*	*Lv*	*Tc*	*Tt*
*Do*	100%						
*Le*	**22.696**	100%					
*Lq*	**35.547**	**18.183**	100%				
*Lr*	**22.867**	**15.322**	**23.182**	100%			
*Lv*	**21.862**	**10.972**	**23.826**	**19.052**	100%		
*Tc*	**25.865**	**36.575**	**47.018**	**31.872**	**36.715**	100%	
*Tt*	**21.964**	**34.154**	**45.713**	**30.121**	**34.482**	**15.201**	100%

When grouped by genera, *Dallinella*, *Laqueus*, and *Terebratalia* each separate along CV1 (Fig 7D). For both CVAs, overall classification accuracy was 100%, with every specimen classified correctly to their genus and species (Tables 4 and 5).

**Table 5:** Mahalanobis distances among genera means for *Dallinella*, *Laqueus*, and *Terebratalia* (bold) and cross-validated classification results in percentages. Overall classification accuracy of 100%.

	*Dallinella*	*Laqueus*	*Terebratalia*
*Dallinella*	100%		
*Laqueus*	**20.610**	100%	
*Terebratalia*	**23.343**	**28.88336**	100%

## Discussion

The results of our study indicate that the traditional approach of identifying and naming terebratulide brachiopod species, emphasizing internal and external morphological characters, remains valid, with individuals of named species clustering together and clearly separating from others in quantitative morphospace. Based only on long loop morphology, all of the species analyzed are statistically different from one another, including those with a problematic taxonomic history such as *L*. *erythraeus/L*. *vancouveriensis* and *T*. *transversa/D*. *occidentalis*.

When analyzing all species together, it was interesting to see that *D*. *occidentalis* and *Terebratalia* separated along the first principal component. This complete separation was not expected since both genera share the same loop type; however, even if specimens of *D*. *occidentalis* have been traditionally placed within the genus *Terebratalia*, their loops are clearly and statistically distinct in shape. In the case of *Laqueus*, every individual was correctly assigned to its named species, even those of *L*. *erythraeus* and *L*. *vancouveriensis*, which had previously been considered geographically distinct subspecies. *Laqueus* species seem to be more morphologically conserved and display low levels of interspecific variability. This was not the case with *Terebratalia*, where there are some erroneous assignments between *T*. *transversa* and *T*. *coreanica*. This result is not surprising given the reported high morphological (ecophenotypic) variability of the genus [45–48], indicating that the ranges of morphological variability of these two species probably overlap, even though they occur on opposite sides of the Pacific Ocean. However, when comparing all of the species together, mean interspecific Mahalanobis distances—a measure of how different two groups are—of *Laqueus erythraeus* and *L*. *vancouveriensis*, and *Terebratalia transversa* and *T*. *coreanica* (15.2 and 15.32, respectively), show that they have similar ranges of intraspecific variability when comparing samples of both East and West Pacific distribution.

Overall, our results suggest that even when external characters are not considered (e.g. foramen size and shape, shell ornamentation and folding, etc.), the loop and cardinalia alone offer sufficient resolution to discriminate among species. In terms of taxonomic classification, our results corroborate the decision to maintain *Laqueus erythraeus* and *Laqueus vancouveriensis* as separate species, as well as keeping *Dallinella occidentalis* separate from *Terebratalia*. *Dallinella* was established as a separate genus based on the fold and sulcus on the valves [53], which are considered to be an important feature in classification [19, 21]. When obtaining samples for this study, we noticed that museum collections often retain the name *Terebratalia occidentalis* for both extant and fossil specimens of *D*. *occidentalis*; when possible, we encourage the revision and update of the material and its respective taxonomic assignments.

### Sample size and phylogenetic structure of our data

Two aspects of our study that require further discussion are the small sample sizes and the lack of known phylogenetic relationships between taxa. The number of individuals analyzed in this study was dependent on multiple factors, mainly the limited availability of specimens in museum collections and the fragility of long loops. Brachiopods are not common components in neontology museum collections and, even when present in larger numbers, long loops are seldom intact due to the delicate nature of these mineralized supporting structures. Museum availability of terebratulide brachiopods is, in turn, influenced by the difficulty associated with collecting—true for many species living in subtidal depths—and commonly small population sizes.

Addressing the effects of phylogenetic structure in our data is complicated since species level relationships in terebratulides remain mostly unexplored. We can make assumptions about some phylogenetic relationships: In *Laqueus*, we can speculate, based in geographic distributions, that *L*. *erythraeus* and *L*. *vancouveriensis* are sister taxa; however, relationships with and among Western Pacific species have not been thoroughly tested. Based on taxonomy, we can assume that *Dallinella* and *Terebratalia* are sister taxa and *Laqueus* the sister taxon of that clade. Although we recognize the importance of analyzing phylogenetically independent data, phylogenetic signal in our data is hard to account for in our analyses since a robust phylogenetic hypothesis is lacking. The addition of DNA sequence data from these species (N. López Carranza and S. Carlson, in prep) will enable species-level phylogenetic analyses to be completed, as well as further testing of species boundaries independent of morphology.

### Methodology and implications for the fossil record

Considering the complexity of long loops, quantifying morphological variation of these structures has been a difficult task. Traditionally, loops have been analyzed using different methods; for example, in living specimens, the mineralized structures and the lophophore they support, can be dissected and examined. Even if the loop remains intact, disarticulating the valves often damages the interlocking hinge structures, usually by breaking the teeth, sockets, and hinge plates, which can be diagnostic characters in species identification. For fossil specimens, destructive techniques such as transverse serial sections have been the most widely used approach to begin to reconstruct the complex loop morphology in three dimensions (e.g. [79–82]). It is possible to use this technique to create 3D models by cutting a fossil into very thin slices perpendicular to the plane of symmetry to produce successive 2D images and then stacking them to generate a 3D reconstruction [83]. Even though this method can analyze samples with low mineralogical density contrast between sedimentary matrix and specimen, it destroys the fossil, which is not ideal for rare specimens.

Given the fragile nature of long loops, these structures are not commonly found intact and unbroken in the fossil record [1], unless they are entombed within lithified sediments inside an individual’s two valves. Unlike bivalved molluscs, with ligaments that open the two valves once the adductor muscles degrade after death, cyrtomatodont brachiopods (i.e. with interlocking hinge teeth and sockets, all terebratulides) can and often do remain articulated after death since contraction of the diductor muscles is required to force the valves open [14]. This creates the possibility that loops can be preserved in fossils, although very difficult to extract in any meaningful way other than by destructive serial sectioning. When possible (when enough contrast exists between the fossil and rock matrix), CT is an excellent high-resolution, non-destructive alternative to serial sectioning.

In paleontology, genera are commonly used as proxies for species, treating them as evolutionary entities, a practice referred to as generification [73]. This practice must be tested with data. Genera are thought to be easier to recognize and distinguish morphologically than species and are thus more easily distinguishable in the fossil record (i.e. traits that identify supraspecific ranks are more generally distributed among individuals and thus more likely to be preserved) [73]. This has been particularly true because few analyses have tested morphological species boundaries in congeneric extant and extinct species of brachiopods.

Macroevolutionary processes are only discerned at the species level and above, and genera and higher taxa are clade-level products of species-level processes. Although analyzing species in paleontological studies can be a demanding task, requiring extensive taxonomic revisions, expertise in systematics, access to and familiarity with large number of specimens, it is essential for understanding morphologic variation, its causes, and evolutionary processes at the species level. Species names and identifications should be treated as hypotheses to be tested, taking into consideration species-delimiting criteria such as morphology, genetics, ecology, and biogeography. To study species in the fossil record, it is important to analyze morphology quantitatively and determine how it varies, since it is the most readily available source of evolutionary information. Moreover, working at the species level also offers a more effective and accessible means of communicating about evolution with researchers from other disciplines, particularly with those studying extant organisms.

## Conclusions

In summary, this study demonstrates that it is possible to discriminate extant species of the genera *Laqueus*, *Terebratalia* and *Dallinella* as statistically distinct entities based on loop and cardinalia morphology. This distinction has been assumed but never before tested quantitatively. Three-dimensional geometric morphometrics offers a robust quantitative approach for testing the morphological validity of these terebratulide brachiopod species that were named solely on the basis of qualitative morphological characters.

## Supporting information

S1 Appendix Accepted species of the genera *Laqueus*, *Terebratalia*, and *Dallinella* (WoRMS, 2019). (DOCX)

S1 Data (TXT)

S2 Data (TXT)

S3 Data (TXT)

S1 Table Specimens analyzed in this study. (DOCX)

S2 Table Landmark and semilandmark (SL) descriptions. (DOCX)
