# Supplementary material for: Testing Species Assignments in Extant Terebratulide Brachiopods: A Three-dimensional Geometric Morphometric Analysis of Long-Looped Brachidia
Source: PLoS One. 2019 Nov 27;14(11):e0225528. doi: 10.1371/journal.pone.0225528 (PMC6881017; doi:10.1371/journal.pone.0225528)
Supplement: S1 Appendix — (DOCX) [file pone.0225528.s001.docx]

Supplementary Materials

S1 Appendix*.* Accepted species of the genera *Laqueus*, *Terebratalia*, and *Dallinella* (WoRMS, 2019)

Phylum Brachiopoda Duméril, 1805

Subphylum Rhynchonelliformea Williams, Carlson, Brunton, Holmer, and Popov, 1996

Class Rhynchonellata Williams, Carlson, Brunton, Holmer, and Popov, 1996

Order Terebratulida Waagen, 1883

Suborder Terebratellidina Muir-Wood, 1955

Superfamily Laqueoidea Thomson, 1927

Family Laqueidae Thomson, 1927

Subfamily Laqueinae Thomson, 1927

Genus *Laqueus* Dall, 1870

*Laqueus astartaeformis* Hatai, 1940 †
*Laqueus blanfordi* (Dunker, 1882)
*Laqueus californicus* (Koch, 1848) (synonymized with Laqueus erythraeus Dall, 1920 by MacKinnon and Long [2000], no longer considered valid)
*Laqueus concentricus* Yabe & Hatai, 1936
*Laqueus erythraeus* Dall, 1920
*Laqueus japonicus* Yabe & Hatai, 1934
*Laqueus jeffreysi* Dall, 1877
*Laqueus morsei* Dall, 1908
*Laqueus orbicularis* Yabe & Hatai, 1934
*Laqueus pacificus* Hatai, 1936
*Laqueus pallidus* Hatai, 1939
*Laqueus pictus* (Chemnitz, 1839)
*Laqueus proprius* Yabe & Hatai, 1934
*Laqueus quadratus* Yabe & Hatai, 1934
*Laqueus rubellus* (Sowerby, 1846)
*Laqueus suffusus* (Dall, 1870)
*Laqueus vancouveriensis* Davidson, 1887

Family Terebrataliidae Richardson, 1975

Subfamily Terebrataliinae Richardson, 1975

Genus *Terebratalia* Beecher, 1893

*Terebratalia coreanica* (Adams & Reeve, 1850)
*Terebratalia gouldi* (Dall, 1891)
*Terebratalia tisimana* Nomura & Hatai, 1936
*Terebratalia transversa* (Sowerby, 1846)

Genus *Dallinella* Thomson, 1915

*Dallinella obsoleta* (Beecher, 1893)
*Dallinella occidentalis* (Dall, 1871)
