# Supplementary material for: Testing Species Assignments in Extant Terebratulide Brachiopods: A Three-dimensional Geometric Morphometric Analysis of Long-Looped Brachidia
Source: PLoS One. 2019 Nov 27;14(11):e0225528. doi: 10.1371/journal.pone.0225528 (PMC6881017; doi:10.1371/journal.pone.0225528)
Supplement: S1 Table — (DOCX) [file pone.0225528.s005.docx]

Supplementary Materials

S1 Table. Specimens analyzed in this study.

| **Specimens** | **Locality** | **Repository** |
| --- | --- | --- |
| *Dallinella occidentalis* SBMNH 467442 | Channel Island, Santa Catalina Island, off White’s Landing; 33° 27’ 57.997’’N, 118° 31’ 27.012’’ | Santa Barbara Museum of Natural History |
| *Dallinella occidentalis* SBMNH 616991 | Channel Island, Santa Catalina Island, off White’s Landing; 33° 27’ 57.997’’N, 118° 31’ 27.012’’ | Santa Barbara Museum of Natural History |
| *Dallinella occidentalis* SBMNH 467448 | Channel Island, Santa Catalina Island, off White’s Landing; 33° 27’ 57.997’’N, 118° 31’ 27.012’’ | Santa Barbara Museum of Natural History |
| *Dallinella occidentalis* SBMNH 616993 | Channel Island, Santa Catalina Island, off White’s Landing; 33° 27’ 57.997’’N, 118° 31’ 27.012’’ | Santa Barbara Museum of Natural History |
| *Dallinella occidentalis* SBMNH 616994 | Channel Island, Santa Catalina Island, off White’s Landing; 33° 27’ 57.997’’N, 118° 31’ 27.012’’ | Santa Barbara Museum of Natural History |
| *Laqueus blanfordi* USNM PAL 716078 | Japan | National Museum of Natural History, Smithsonian Institution |
| *Laqueus erythraeus* CAS IZ 202358A | Channel Island, Gulf of Santa Catalina, 33.00° 20.80’ N, 18° 18.50’ W, depth: 79-62 m. | California Academy of Sciences |
| *Laqueus erythraeus* CAS IZ 202358B | Channel Island, Gulf of Santa Catalina, 33.00° 20.80’ N, 18° 18.50’ W, depth: 79-62 m. | California Academy of Sciences |
| *Laqueus erythraeus* CAS IZ 202358C | Channel Island, Gulf of Santa Catalina, 33.00° 20.80’ N, 18° 18.50’ W, depth: 79-62 m. | California Academy of Sciences |
| *Laqueus erythraeus* CAS IZ 202358D | Channel Island, Gulf of Santa Catalina, 33.00° 20.80’ N, 18° 18.50’ W, depth: 79-62 m. | California Academy of Sciences |
| *Laqueus erythraeus* CAS IZ 202358E | Channel Island, Gulf of Santa Catalina, 33.00° 20.80’ N, 18° 18.50’ W, depth: 79-62 m. | California Academy of Sciences |
| *Laqueus erythraeus* CAS IZ 202358F | Channel Island, Gulf of Santa Catalina, 33.00° 20.80’ N, 18° 18.50’ W, depth: 79-62 m. | California Academy of Sciences |
| *Laqueus erythraeus* DAV:SJCLab 0001 | Monterey Bay, California. | Carlson Lab Collections, UCD |
| *Laqueus erythraeus* DAV:SJCLab 0002 | Monterey Bay, California | Carlson Lab Collections, UCD |
| *Laqueus erythraeus* DAV:SJCLab 0003 | Monterey Bay, California | Carlson Lab Collections, UCD |
| *Laqueus erythraeus* DAV:SJCLab 0004 | Monterey Bay, California | Carlson Lab Collections, UCD |
| *Laqueus erythraeus* DAV:SJCLab 0005 | Monterey Bay, California | Carlson Lab Collections, UCD |
| *Laqueus erythraeus* DAV:SJCLab 0006 | Monterey Bay, California | Carlson Lab Collections, UCD |
| *Laqueus erythraeus* DAV:SJCLab 0007 | Monterey Bay, California | Carlson Lab Collections, UCD |
| *Laqueus erythraeus* DAV:SJCLab 0008 | Monterey Bay, California | Carlson Lab Collections, UCD |
| *Laqueus erythraeus* DAV:SJCLab 0009 | Monterey Bay, California | Carlson Lab Collections, UCD |
| *Laqueus erythraeus* DAV:SJCLab 0010 | Monterey Bay, California | Carlson Lab Collections, UCD |
| *Laqueus quadratus* USNM PAL 716076 | Kii, Japan | National Museum of Natural History, Smithsonian Institution |
| *Laqueus quadratus* USNM PAL 716077 | Kii, Japan | National Museum of Natural History, Smithsonian Institution |
| *Laqueus rubellus* USNM PAL 716067 | Kagoshima Gulf, Japan | National Museum of Natural History, Smithsonian Institution |
| *Laqueus rubellus* USNM PAL 716068 | Kagoshima Gulf, Japan | National Museum of Natural History, Smithsonian Institution |
| *Laqueus rubellus* USNM PAL 716069 | Kagoshima Gulf, Japan | National Museum of Natural History, Smithsonian Institution |
| *Laqueus rubellus* USNM PAL 716070 | Kagoshima Gulf, Japan | National Museum of Natural History, Smithsonian Institution |
| *Laqueus rubellus* USNM PAL 716071 | Kagoshima Gulf, Japan | National Museum of Natural History, Smithsonian Institution |
| *Laqueus rubellus* USNM PAL 716072 | Kagoshima Gulf, Japan | National Museum of Natural History, Smithsonian Institution |
| *Laqueus rubellus* USNM PAL 716073 | Kagoshima Gulf, Japan | National Museum of Natural History, Smithsonian Institution |
| *Laqueus rubellus* USNM PAL 716074 | Kagoshima Gulf, Japan | National Museum of Natural History, Smithsonian Institution |
| *Laqueus rubellus* USNM PAL 716075 | Kagoshima Gulf, Japan | National Museum of Natural History, Smithsonian Institution |
| *Laqueus vancouveriensis* USNM PAL 716055 | 50° 49’ N, 127° 36’ 30’’ W, off NW of Port Alice, British Columbia, Canada | National Museum of Natural History, Smithsonian Institution |
| *Laqueus vancouveriensis* USNM PAL 716056 | 50° 49’ N, 127° 36’ 30’’ W, off NW of Port Alice, British Columbia, Canada | National Museum of Natural History, Smithsonian Institution |
| *Laqueus vancouveriensis* USNM PAL 716057 | 50° 49’ N, 127° 36’ 30’’ W, off NW of Port Alice, British Columbia, Canada | National Museum of Natural History, Smithsonian Institution |
| *Laqueus vancouveriensis* USNM PAL 716058 | 50° 49’ N, 127° 36’ 30’’ W, off NW of Port Alice, British Columbia, Canada | National Museum of Natural History, Smithsonian Institution |
| *Laqueus vancouveriensis* USNM PAL 716059 | 50° 49’ N, 127° 36’ 30’’ W, off NW of Port Alice, British Columbia, Canada | National Museum of Natural History, Smithsonian Institution |
| *Laqueus vancouveriensis* USNM PAL 716060 | 50° 49’ N, 127° 36’ 30’’ W, off NW of Port Alice, British Columbia, Canada | National Museum of Natural History, Smithsonian Institution |
| *Laqueus vancouveriensis* USNM PAL 716061 | 50° 49’ N, 127° 36’ 30’’ W, off NW of Port Alice, British Columbia, Canada | National Museum of Natural History, Smithsonian Institution |
| *Laqueus vancouveriensis* USNM PAL 716062 | 50° 49’ N, 127° 36’ 30’’ W, off NW of Port Alice, British Columbia, Canada | National Museum of Natural History, Smithsonian Institution |
| *Laqueus vancouveriensis* USNM PAL 716063 | 50° 49’ N, 127° 36’ 30’’ W, off NW of Port Alice, British Columbia, Canada | National Museum of Natural History, Smithsonian Institution |
| *Laqueus vancouveriensis* USNM PAL 716064 | 50° 49’ N, 127° 36’ 30’’ W, off NW of Port Alice, British Columbia, Canada | National Museum of Natural History, Smithsonian Institution |
| *Laqueus vancouveriensis* USNM PAL 716065 | 50° 49’ N, 127° 36’ 30’’ W, off NW of Port Alice, British Columbia, Canada | National Museum of Natural History, Smithsonian Institution |
| *Laqueus vancouveriensis* USNM PAL 716066 | 50° 49’ N, 127° 36’ 30’’ W, off NW of Port Alice, British Columbia, Canada | National Museum of Natural History, Smithsonian Institution |
| *Terebratalia coreanica* USNM PAL 716050 | Hokodate, Hokkaido, Japan | National Museum of Natural History, Smithsonian Institution |
| *Terebratalia coreanica* USNM PAL 716051 | Hokodate, Hokkaido, Japan | National Museum of Natural History, Smithsonian Institution |
| *Terebratalia coreanica* USNM PAL 716052 | Hokodate, Hokkaido, Japan | National Museum of Natural History, Smithsonian Institution |
| *Terebratalia coreanica* USNM PAL 716053 | Hokodate, Hokkaido, Japan | National Museum of Natural History, Smithsonian Institution |
| *Terebratalia coreanica* USNM PAL 716054 | Hokodate, Hokkaido, Japan | National Museum of Natural History, Smithsonian Institution |
| *Terebratalia transversa* DAV:SJCLab 0011 | Friday Harbor, WA | Carlson Lab Collections, UCD |
| *Terebratalia transversa* DAV:SJCLab 0012 | Friday Harbor, WA | Carlson Lab Collections, UCD |
| *Terebratalia transversa* DAV:SJCLab 0013 | Friday Harbor, WA | Carlson Lab Collections, UCD |
| *Terebratalia transversa* SBMNH 467446 | 47° 16’ 30.000’’N, 122° 32’ 45.000’’ W, Pierce County, Tacoma Narrows, WA, intertidal | Santa Barbara Museum of Natural History |
| *Terebratalia transversa* SBMNH 616986 | 47° 16’ 30.000’’ N, 122° 32’ 45.000’’ W, Pierce County, Tacoma Narrows, WA, intertidal | Santa Barbara Museum of Natural History |
| *Terebratalia transversa* SBMNH 616988 | 47° 16’ 30.000’’ N, 122° 32’ 45.000’’ W, Pierce County, Tacoma Narrows, WA, intertidal | Santa Barbara Museum of Natural History |
| *Terebratalia transversa* SBMNH 616989 | 47° 16’ 30.000’’ N, 122° 32’ 45.000’’ W, Pierce County, Tacoma Narrows, WA, intertidal | Santa Barbara Museum of Natural History |
| *Terebratalia transversa* SBMNH 616990 | 47° 16’ 30.000’’ N, 122° 32’ 45.000’’ W, Pierce County, Tacoma Narrows, WA, intertidal | Santa Barbara Museum of Natural History |
