# Supplementary material for: Testing Species Assignments in Extant Terebratulide Brachiopods: A Three-dimensional Geometric Morphometric Analysis of Long-Looped Brachidia
Source: PLoS One. 2019 Nov 27;14(11):e0225528. doi: 10.1371/journal.pone.0225528 (PMC6881017; doi:10.1371/journal.pone.0225528)
Supplement: S2 Table — (DOCX) [file pone.0225528.s006.docx]

Supplementary Materials

S2 Table. Landmark and semilandmark (SL) descriptions.

| Landmarks | Description | Landmark type | Loop type |
| --- | --- | --- | --- |
| 1 | Posterior edge of cardinal process | Type II | Bilateral/trabecular |
| 2 | Lateral edge of cardinal process | Type II | Bilateral/trabecular |
| 3 | Anterior tip of cardinal process | Type II | Bilateral/trabecular |
| 4 | Junction of crus and inner hinge plate | Type I | Bilateral/trabecular |
| 5 | Tip of crural process | Type II | Bilateral/trabecular |
| 6 | Junction of descending and ascending branches | Type I | Bilateral/trabecular |
| 7 | Junction of crus and outer hinge plate | Type I | Bilateral/trabecular |
| 8 | Junction of inner socket ridge and socket | Type I | Bilateral/trabecular |
| 9 | Maximal curvature of socket | Type II | Bilateral/trabecular |
| 10 | Junction of outer socket ridge and dorsal valve | Type I | Bilateral/trabecular |
| 11 | Posterior edge of transverse band | Type II | Bilateral/trabecular |
| 12 | Anterior edge of transverse band | Type II | Bilateral/trabecular |
| 13 | Junction of horizontal connecting band and septum | Type I | Bilateral/trabecular |
| 14 | Ventral junction of horizontal connecting band and descending branch | Type I | Bilateral/trabecular |
| 15 | Dorsal junction of horizontal connecting band and descending branch | Type I | Bilateral/trabecular |
| 16 and 17 | Ventral junctions of vertical connecting band and descending branch | Type I | Bilateral |
| 18 and 19 | Dorsal junctions of vertical connecting band and ascending branch | Type I | Bilateral |
| 20 | Junction of inner hinge plate and septum | Type I | Bilateral |
| Semilandmarks | Description | Landmark type | Loop type |
| Curve 1 (1 SL) | Crus (from landmark 4 to 5) | SL | Bilateral/trabecular |
| Curve 2 (11 SL) | Descending branch (from landmark 5 to 6) | SL | Bilateral/trabecular |
| Curve 3 (9 SL) | Ascending branch (from landmark 6 to 12) | SL | Bilateral/trabecular |
